# The Effect of Parental Medical History on the Prevalence of Cerebrovascular Diseases in Their Children in an Iranian Population

**DOI:** 10.32598/bcn.9.5.367

**Published:** 2018-09-01

**Authors:** Alireza Khosravi, Mohaddeseh Behjati, Minoo Dianatkhah, Fatemeh Noori, Nizal Sarrafzadegan, Majid Nejati

**Affiliations:** 1. Hypertension Research Center, Cardiovascular Research Institute, Isfahan University of Medical Sciences, Isfahan, Iran.; 2. Interventional Cardiology Research Center, Cardiovascular Research Institute, Isfahan University of Medical Sciences, Isfahan, Iran.; 3. Heart Failure Research Center, Cardiovascular Research Institute, Isfahan University of Medical Science, Isfahan, Iran.; 4. Anatomical Sciences Research Center, Kashan University of Medical Sciences, Kashan, Iran.

**Keywords:** Parental, Medical history, Cerebrovascular, Children

## Abstract

**Introduction:**

Still a controversial issue, family history is known as a risk factor for the development of Cerebrovascular Diseases (CVD). In this study, we aimed to evaluate the relationship between parental history and risk of CVD in their offspring in Iranian population.

**Methods:**

Isfahan Cohort Study (ICS) included total 6504 healthy participants which were randomly selected through a two-stage cluster sampling method from three districts. The participants were followed prospectively for 10 years. The diagnosis of CVD were confirmed by expert panelist. Clinically validated history of CVD was established for definition of parental history of CVD. Types of history were categorized into paternal, maternal, both parents, and no history.

**Results:**

The prevalence of CVD is generally higher among female offspring compared with male ones (P<0.001). The relative risk of CVD with maternal history was not significant (95%CI=0.95–2.29). By adjusted model analysis, history of CVD in both parents affected the risk of CVD in their male children (RR=2.13, P=0.033, 95%CI). By crude model analysis, maternal history of CVD (P=0.047), history of CVD in both parents (P=0.032), and maternal history of hypertension (P=0.005) were determined as risk factors of CVD in offspring. Indeed, the mean age of CVD in offspring decreases based on this order: history of hypertension in parents, paternal history of CVD in both parents, maternal history of CVD, and no history (P<0.001).

**Conclusion:**

Early and regular screening for CVD development is necessary in female offspring of the families with the present history of CVD from maternal side. This group are at risk and should be considered as the target group for screening and taking preventive measures.

## Highlights

- Maternal transmission of cerebrovascular diseases imposes a greater risk on their offspring.
- Women with a maternal history of cerebrovascular disease are more susceptible to develop cerebrovascular diseases.
- Age of cerebrovascular disease onset in patients with positive family history is lower than the normal population.

## Plain Language Summary

The role of genetics in Cerebrovascular Diseases (CVDs) is not obvious. Traditional risk factors such as hypertension, diabetes mellitus, hyperlipidemia, obesity and smoking association with parental history of CVD were investigated. Maternal history of CVD, history of CVD in both parents and maternal history of hypertension were determined as risk factors of CVD in offspring. This study results indicate that the prevalence of CVD in female offspring of people with a positive CVD history is more than male offspring. The onset age of CVD in offspring decreases based on this order as history of hypertension in parents, paternal history of CVD in both parents, maternal history of CVD, and no history. It seems essential that earlier and regular screening of CVD be done in female offspring with maternal history of CVD.

## 1. Introduction

Cerebrovascular Diseases (CVDs) are multi-factorial diseases, caused by environmental factors and poor lifestyle choices such as poor diet, smoking and other predisposing diseases. Although the role of genetics in CVD has been already discussed, the effect of parental history of CVD on offspring CVD risk is unclear (Seshadri et al., 2010). Numerous studies explored the relationship between family history and risk for stroke (Chung et al., 2016; Tian et al., 2017).

Such family history has been reported by various studies on twin and animal models (Jood, Ladenvall, Rosengren, Blomstrand, & Jern, 2005; Aparicio & Seshadri, 2017). CVD has a substantial familial aggregation and heritability in certain families that result in its earlier development (Bak, Gaist, Sindrup, Skytthe, & Christensen, 2002; Seshadri et al., 2006). The risk of stroke in middle-aged to old-aged people is partly associated with a family history of stroke. This familial history provokes the risk for both hemorrhagic and ischemic stroke and post hospital discharge neurobehavioral outcomes, especially in young women (Kim et al., 2004).

Parental history of Coronary Heart Disease (CHD) is an obvious risk factor for the occurrence of disease in the offspring. However, clear correlation has not been established between CVD and family history yet (Sholtz, Rosenmanr, & Brandr, 1975; Jousilahti, Puska, Vartiainen, Pekkanen, & Tuomilehto, 1996). It has been demonstrated that positive family history of stroke is associated with increased risk of stroke in females compared with males with more association from maternal side (Tentschert, Greisenegger, Wimmer, Lang, & Lalouschek, 2003; Seshadri et al., 2010). The cause of such correlation is unknown; however, genetic factors seem to have the main role (Huang et al., 1994).

According to Floßmann, Schulz and Rothwell (2004) the impact of family history on CVD in offspring is very heterogeneous. This heterogeneity could be attributed to different study populations, insufficient details, or research bias (Øygarden et al., 2015). Thus, it seems necessary to entirely determine the inheritance pattern of this disease for each population, to provide better preventive, diagnostic, and therapeutic options for at-risk subgroups. Understanding the detailed family history of CVD with special attention to sex differences can help develop preventive programs or risk assessment prediction models. Therefore, this study aimed to evaluate and compare the impact of maternal and paternal history of CVD on the risk of CVD development in the offspring of study population.

## 2. Methods

Isfahan Cohort Study (ICS) is a 10-year longitudinal prospective and ongoing population-based study, which began in 2001 (January 2 to September 28) (Sarrafzadegan et al., 2011). ICS followed up 6504 individuals, aged 35 years or more. In total, 51% of the participants were women. The samples were selected from rural and urban areas in 3 districts, including Najafabad, Arak and Isfahan cities, Iran. Of them, 6323 were initially free from CVD. According to WHO definition, stroke is a rapid-onset focal neurological disorder with probable vascular cause lasting more than 24 hours. A combination of stroke and IHD was used to define CVD.

The final diagnosis was made by a panel of neurologists and cardiologists based on medical records, while verbal autopsy was conducted for deaths in the follow-up (Sarrafzadegan, Sadeghi, Oveisgharan, & Iranipour, 2013). Self-reported history of CVD was used to define parental history of CVD. Types of parental history of CVD were categorized as: no parental history of CVD, both parental history of CVD or Hypertension (HTN).

For statistical analysis, the relative risk and 95% Confidence Interval (CI) of HTN, Diabetes Mellitus (DM), hyperlipidemia, obesity and smoking and association of these risk factors with parental history of CVD were investigated. All participants who had CVD during follow-up were selected as the case group and those who did not were selected as the control group. Both groups were matched in time of follow-up (density sampling), so that the effect of time on the risk was similar.

Statistical analyses were performed using SPSS. Multivariable analysis was performed to determine effect of parental history of hypertension on occurrence of hypertension in their offspring. Both crude and adjusted model were applied for analysis of data. Adjustment was performed based on age, gender and Body Mass Index (BMI) of offspring.

## 3. Results

The study results indicated that the overall prevalence of CVD in female offspring of people with a positive CVD history is more than male offspring (P<0.001). Table 1 demonstrates the gender-specific distribution of CVD with respect to the parental history. Table 1 also presents risk factors and demographic data regarding CVD in the offspring. According to crude analysis, DM (3.07, CI: 2.49–3.79), age older than 60 years (3.04, CI=2.54–3.63), BMI>30 (1.25, CI=1.02–1.53), and female gender (0.78, CI: 0.66–0.93) were significantly related with CVD in offspring. Adjusted model revealed that, age older than 60 years (3.22, CI=2.68–3.85), DM (1.76, CI=1.76–2.80), BMI>30 (1.49, CI=1.21–1.84), maternal history of HTN (1.45, CI=1.11–1.89), female gender (0.70, CI=0.58–0.84), and history of CVD alone in both parents influenced the risk for CVD in male off-spring (RR=2.13, P=0.033, 95%CI).

**Table 1.** The relationship between CVD risk factors and family history of participants based on parental history

**Offspring Variable**	**No History (n=434)**	**Father or Mother (n=506)**	**P**	**Father (n=297)**	**Mother (n=209)**	**Both (n=51)**	**P**
Sex (Male)	196(45.2)	243(43.6)	<0.001	156(52.5)	71(34.0)	16(31.4)	<0.001
Age at baseline	49.3±10.9	47.6±9.5	0.010	47.0±9.51	48.4±9.74	47.8±8.95	0.281
BMI	26.9±4.52	27.6±4.46	0.020	27.4±4.28	27.8±4.70	28.1±4.48	0.386
Smoking	90(20.7)	117(21.1)	0.895	71(24.1)	38(18.2)	8(15.7)	0.171
Diabetes	54(12.4)	60(10.8)	0.414	26(8.8)	26(12.4)	8(15.7)	0.208
Hypertension	134(30.9)	158(28.4)	0.390	73(24.6)	68(32.5)	17(33.3)	0.105
Low-HDL	198(45.6)	264(47.4)	0.578	137(46.1)	104(49.8)	23(45.1)	0.681
High-LDL	204(47.0)	267(47.9)	0.771	132(44.4)	113(54.1)	22(43.1)	0.079
High Cholesterol	252(58.1)	332(59.6)	0.625	165(55.6)	137(65.6)	30(58.8)	0.078
High Triglyceride	251(57.8)	357(64.1)	0.045	194(65.3)	127(60.8)	36(70.6)	0.344

Data are presented as Mean±SD or No. (%).

Although the relative risk of maternal history of CVD was higher than paternal history, the difference was not significant (CI=0.95–2.29). Presence or absence of parental history of HTN (each type) does not influence the development of CVD in offspring. By crude model analysis, maternal history of CVD (P=0.047), history of CVD in both parents (P=0.032), maternal history of HTN (P=0.005), as well as history of DM (P<0.001) and smoking (P=0.026) were determined as risk factors for the occurrence of CVD in offspring.

Female gender (P=0.005), age of more than 60 years (P<0.001) and obesity (BMI>30) (P=0.028) makes off-spring more susceptible to CVD. Furthermore, high total cholesterol levels (>200 mg/dL) (P=0.62), high serum level of LDL (>150 mg/dL) (P=0.77) and low HDL (<30 mg/dL) (P=0.57) did not affect the risk for CVD in offspring. While a high triglyceride level (>200 mg/dL) (P=0.045) relatively impacts the risk for CVD in off-spring (Table 1).

Only maternal history of HTN has a significant effect on the incidence of CVD. Nevertheless, adjusted analysis for gender, age and BMI clarified that the age of CVD incidence in patients with positive family history is lower than the normal population (57.9±10.9 vs. 61.9±11.1 years, P=0.031) (Table 2). However, there were no significant differences between paternal and maternal family history in the onset age of CVD in the offspring (P=0.835). Moreover, the mean age of CVD incidence in offspring with paternal, maternal and both history of HTN is significantly lower than the group without parental history of HTN (P=0.013).

**Table 2.** The hazard ratio of CVD based on family history

**Models**	**Family History**	**HR (95%CI)**	**P**	**HR (95%CI)**	**P**
Crude	None			1	
	Paternal	0.47(0.25–0.89)	0.021	0.96(0.64–1.45)	0.843
	Maternal	0.72(0.38–1.36)	0.318	1.46(0.97–2.21)	0.068
	Both	1	-	1.98(1.09–3.61)	0.025
Model 1	None			1	
	Paternal	0.46(0.24–0.87)	0.017	0.96(0.63–1.44)	0.838
	Maternal	0.72(0.38–1.36)	0.311	1.47(0.97–2.22)	0.068
	Both	1	-	1.99(1.09–3.62)	0.025
Model 2	None			1	
	Paternal	0.48(0.25–0.91)	0.024	1.11(0.73–1.68)	0.624
	Maternal	0.70(0.37–1.32)	0.268	1.56(1.03–2.35)	0.036
	Both	1	-	1.20(1.21–4.01)	0.010
Model 3	None			1	
	Paternal	0.48(0.25–0.92)	0.026	1.05(0.69–1.59)	0.817
	Maternal	0.69(0.36–1.30)	0.251	1.41(0.93–2.15)	0.104
	Both	1	-	2.05(1.12–3.74)	0.019

Paternal and maternal history were compared to both parental history (left column) and none-familial history (right column); Data adjusted in Model 1 with: Gender, Model 2: Age and Gender and Model 3: Age, Gender and BMI.

## 4. Discussion

The obtained results indicated that women with a maternal history of CVD are more susceptible to CVD. Thus maternal side history seems to be a risk factor which needs regular screening for CVD. In addition to a positive parental history, some risk factors in offspring such as DM, age of more than 60 years, female gender and obesity (BMI>30) are associated with higher risk of CVD. Such characteristics could be explained by the impact of conventional risk factors for CVD in the general population. These factors in addition to maternal history of HTN and just history of both parents are associated with slightly greater CVD risk in male offspring. The accumulation of these risk factors together in the family may lead to the occurrence of CVD in the earlier ages of offspring.

Several studies have addressed the association between family history and CVD (Barrett-Connor & Khaw, 1984; Hunt, Gwinn, & Adams, 2003; Woodward, Brindle, & Tunstall-Pedoe, 2007). Prospective, community-based studies, and two-generation Framingham study cohort reported that the impact of parental stroke was greatest on early stroke incidence, before the age of 65 years. Genetic epidemiology of stroke is relatively unexplored and gender-specific aspects of this relationship are poorly explained (McBride, Hale, Subramanian, Badger, & Bernstein, 2014). The results of this investigation demonstrated marked gender-specific differences of the relationship between paternal and or maternal history of CVD and the occurrence of CVD in offspring.

Our data indicated that maternal transmission of CVD imposes a greater risk on offspring, especially in females. Despite the lack of consensus on the impact of family history on CVD in offspring, our results is consistent with previous studies (Hunt et al., 2003; Qureshi et al., 2012). The incidence of CVD in women is more affected than men by parental history of the disease. Therefore, parental history is a better predictor for female offspring. This finding is consistent with a previous study that indicated a gender-specific relationship between maternal history of CVD, left ventricular hypertrophy and HTN in female patients with CVD events (Tentschert et al., 2003).

Extensive assessment of family history in development of CVD among offspring’s maternal or paternal grandfathers, grandmothers and other relatives may be helpful for better stratification of risk factors in offspring. The risk for CVD in female offspring with positive family history of CVD is higher. But it should be kept in mind that this higher conferred risk could not be introduced to the person as a cause of life-long stress. These data should be applied correctly for screening at risk population for primary prevention. In order to accurately assess inheritance traits in children, the environmental factors and lifestyle in the family should also be taken into account.

Family history impacts the chance of catching CVD in the offspring. It is necessary to conduct initial and regular screening of CVD, in female offspring with maternal history of CVD. This susceptible population should be targeted for screening and preventive measures.

## Ethical Considerations

### Compliance with ethical guidelines

All methods of this study were approved by Local Ethic Committee (Code: 82112).
